# A Woman with Abdominal Pain

**DOI:** 10.5811/cpcem.43521

**Published:** 2025-06-08

**Authors:** Colton Conrad, Reginald Alouidor, Christopher Allison

**Affiliations:** * University of Massachusetts Medical School – TH Chan School of Medicine, Baystate Medical Center Department of Emergency Medicine, Springfield, Massachusetts; † University of Massachusetts Medical School – TH Chan School of Medicine, Baystate Medical Center Department of Critical Care Medicine, Springfield, Massachusetts; ‡ University of Massachusetts Medical School – TH Chan School of Medicine, Baystate Medical Center Division of Trauma/Critical Care and Acute Care Surgery, Springfield, Massachusetts

**Keywords:** intussusception, colectomy, gastroenterology, polysubstance use

## Abstract

**Case Presentation:**

A 28-year-old woman with a history of cocaine and opioid use disorder presented to the emergency department with abdominal pain, nausea, and vomiting for two days. She’d had irregular bowel movements with constipation for quite some time. Physical exam was notable for diffuse peritonitis and melena on digital rectal exam. Patient had a witnessed episode of hematochezia. Computed tomography of the abdomen and pelvis with intravenous contrast demonstrated sigmoid colon intussusception, and the patient underwent emergent surgery for definitive treatment. Specimen was sent to surgical pathology and revealed no lead point.

**Discussion:**

While sigmoid intussusception is not a rare finding, it is exceedingly rare in young adult patients who do not have a pathologic lead point. Lead points are areas of inflammation, lesions, or masses that snag the bowel and initiate the process of telescoping that ultimately results in an intussusception. This patient was not found to have such a lead point on gross examination during surgery or on extensive specimen examination in the pathology lab. Instead, her sigmoid intussusception is hypothesized to be secondary to decreased gut motility in the setting of chronic opioid use disorder.

## CASE PRESENTATION

**Image 1:**
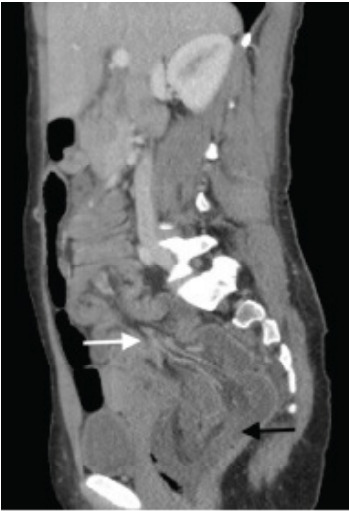
Sagittal computed tomography view showing edematous sigmoid colon (black arrow) containing omentum (white arrow).

**Image 2:**
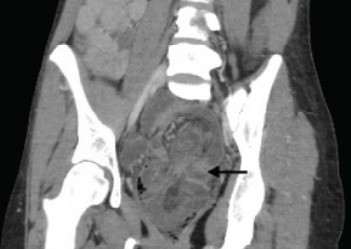
Coronal computed tomography view showing edematous sigmoid colon containing intussuscepted descending colon (black arrow).

**Image 3:**
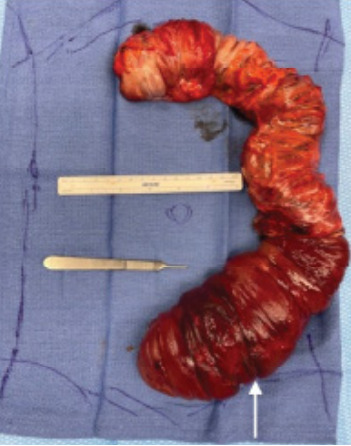
Excised sigmoid, descending, and transverse colon, showing stacked layers of bowel within the sigmoid colon with ischemic necrosis and hemorrhagic walls (white arrow).

A 28-year-old woman with a history of cocaine and heroin use presented with abdominal pain, nausea, and vomiting worsening for two days. On physical exam, the patient was noted to have peritonitis in all four quadrants of the abdomen with absent bowel sounds and melanotic stool on digital rectal exam, as well as a witnessed episode of hematochezia. Vital signs were significant for tachycardia. Laboratory studies were significant only for a leukocytosis of 13.1 x 10^3^/liter (L) (reference range: 4.5–11.0 x 10^3^/L). The patient received antibiotics and antiemetic therapy, as well as pain control. Computed tomography (CT) of the abdomen and pelvis with intravenous (IV) contrast revealed the diagnosis of intussusception involving the sigmoid colon, and the transverse and descending colon, as well as part of the omentum (Images 1 and 2). She underwent emergency surgery for definitive treatment (Image 3).

## DISCUSSION

Computed tomography with IV contrast demonstrated sigmoid colon intussusception involving the transverse and descending colon, as well as parts of the omentum with evidence of ischemic necrosis. The patient underwent emergent sigmoidectomy, sub-total colectomy, partial omentectomy, and creation of end-ileostomy. The resected specimen was evaluated by pathology but demonstrated no lead point. Our patient had an extensive history of constipation, likely secondary to her opioid dependence. Opioids act on the mu opioid receptors within the small and large bowel, leading to decreased smooth muscle contractions, diminished colonic peristalsis, and prolonged transit times.^1^ We hypothesize chronic opioid use contributed to her intussusception. Only one other case report in recent literature describes a patient with intussusception without pathologic lead point in the setting of chronic cocaine and opioid dependence.^2^ The prevalence of fentanyl contamination in cocaine and methamphetamine is estimated to be greater than 10%.^3^ Thus, patients who primarily use stimulants may unknowingly consume opioids on a regular basis, putting them at risk for decreased gut motility.

CPC-EM Capsule

What do we already know about this clinical entity?

*Intussusception is relatively rare in adults and is generally associated with older age, adhesions from prior surgeries, inflammatory bowel disease, or malignancy*.

What is the major impact of the image(s)?

*The combination of computed tomography images and gross specimen photograph help the reader correlate typical radiographic findings with the physiologic process*.

How might this improve emergency medicine practice?

*This case serves as a reminder to consider atypical presentations of intussusception especially in young patients with risk factors such as chronic opioid use*.

Intussusception in adults is rare, with an incidence of 2–3 cases per million annually, and comprising only 5% of all cases. Approximately 80–90% of adult intussusception cases extend from pathologic lead points that may represent inflammatory bowel disease, diverticular disease, polyps, or malignancy.^4^ The remaining 10–20% of intussusception cases without a pathologic lead point are termed “idiopathic.” The diagnostic modality of choice is CT with IV contrast, which may elucidate an existing lead point. Definitive treatment is resection via laparotomy, which exposes the entire colon for evaluation.^5^
